# Disparities in Health Insurance Among Middle Eastern and North African American Children in the US

**DOI:** 10.1177/21501319241255542

**Published:** 2024-05-20

**Authors:** Florence J. Dallo, Kyrah K. Brown, Adebola Obembe, Tiffany Kindratt

**Affiliations:** 1 Oakland University, Rochester, MI, USA; 2 University of Texas at Arlington, Arlington, TX, USA

**Keywords:** health insurance, Middle Eastern and North African, children, National Health Interview Survey, American Community Survey

## Abstract

**Objective:**

To estimate and compare the proportion of foreign-born Middle Eastern/North African (MENA) children without health insurance, public, or private insurance to foreign- and US-born White and US-born MENA children.

**Methods:**

Using 2000 to 2018 National Health Interview Survey data (N = 311 961 children) and 2015 to 2019 American Community Survey data (n = 1 892 255 children), we ran multivariable logistic regression to test the association between region of birth among non-Hispanic White children (independent variable) and health insurance coverage types (dependent variables).

**Results:**

In the NHIS and ACS, foreign-born MENA children had higher odds of being uninsured (NHIS OR = 1.50, 95%CI = 1.10-2.05; ACS OR = 2.11, 95%CI = 1.88-2.37) compared to US-born White children. In the ACS, foreign-born MENA children had 2.11 times higher odds (95%CI = 1.83-2.45) of being uninsured compared to US-born MENA children.

**Conclusion:**

Our findings have implications for the health status of foreign-born MENA children, who are currently more likely to be uninsured. Strategies such as interventions to increase health insurance enrollment, updating enrollment forms to capture race, ethnicity, and nativity can aid in identifying and monitoring key disparities among MENA children.

## Introduction

Among children 0 to 17 years old, health insurance coverage estimates have been periodically reported by United States (US) federal agencies. In 2021, 4.1% of children were uninsured, 44.3% had public health insurance coverage, and 53.8% had private health insurance coverage. As a result of structural and socioeconomic barriers and inequities,^1^ health insurance coverage estimates among children vary by race, ethnicity, and nativity status. In 2021, Hispanic children had higher rates of being uninsured (7.8%), followed by non-Hispanic Black (3.0%), non-Hispanic White (2.7%), and Asian (1.3%) children. Public health insurance was most common among non-Hispanic Black (66.1%) and Hispanic (61.2%) children compared to non-Hispanic White (31.5%) and Asian (20.4%) children.^2^ Private health insurance was most common among Asian (74.1%) and non-Hispanic White (68.5%) children compared to non-Hispanic Black (32.6%) and Hispanic (32.5%) children.^2^ Also, US-born children had lower rates (7.76%) of not having insurance compared to immigrant children (32.16%).^3^

Population health research focused on disaggregating groups by race, ethnicity, and nativity is crucial for unmasking significant health disparities that may exist within broadly defined racial and ethnic groups.^4^ In the US, individuals classified as non-Hispanic White are defined as having origins in “any of the original peoples of Europe, the Middle East, or North Africa” (MENA).^5^ Available data indicate that non-Hispanic White immigrant children were more likely to be uninsured (13.6%) compared to US-born White children (5.8%).^3^ People classified as “non-Hispanic White” represent a heterogenous group and prior studies have documented within-group disparities.^6^ While relatively few studies in the maternal and child health field have examined disparate health and healthcare outcomes among MENA,^7-9^ there are currently no published studies examining health insurance coverage among MENA children.

National research describing health insurance coverage estimates for MENA children is necessary for several reasons. First, health insurance is an important predictor of health care access, use, and quality.^10^ Second, health insurance influences the diagnoses, treatment and prognosis of health conditions, vaccination uptake, and other factors.^11^ Third, the MENA population has grown significantly in the US in the last 30 years, and little is known about the health and wellbeing of MENA children, especially by nativity status.^7^-^9^ Lastly, there are only two2 national surveys where health insurance coverage for MENA children can be measured: the National Health Interview Survey (NHIS) and the American Community Survey (ACS). We will leverage the strengths of these surveys to provide valid and reliable estimates of health insurance coverage among MENA children.

Given these issues, this study has two aims. The first aim is to estimate and compare the proportion of foreign-born MENA children without health insurance to foreign- and US-born White children. We hypothesize that the proportion of foreign-born MENA children *without* health insurance will be greater than the other two groups. Also, we hypothesize the odds of *not* having any health insurance coverage will be greater among foreign-born MENA children than the other two groups. The second aim is to estimate and compare the proportion of foreign-born MENA children without health insurance to US-born MENA children.

## Methods

### Sample

The sample was selected from the National Health Interview Survey (NHIS; 2008-2018). The NHIS uses a complex sample design to conduct annual household surveys that collect health, demographic, and socioeconomic status information from all individuals in the household. One child per family (<17 years old) is randomly selected as a sample child, where an adult in the family that has the most knowledge about the child’s health responds to the questions.^12^ The sample child section includes topics such as health conditions, limitations of activity, health status, health care access and utilization, mental health, and influenza vaccination. Additional details of the NHIS are provided elsewhere.^12^ In this study, the NHIS sample included 311 961 children, of which 305 454 were US-born whites, 4738 foreign-born whites, and 1769 were foreign-born MENA.

We also analyzed 2015 to 2019 ACS data. The ACS collects monthly samples to produce annual national estimates of demographic and socioeconomic factors.^13^ A random sample of addresses are selected each year. The sampling design is structured so that each household should not be selected more than once over a five-year period.^13^ While the ACS does not directly measure personal health information (ie, health behaviors and conditions), it measures health insurance coverage. The ACS collects data using multiple data collection modalities, including mailing, phone interviews and personal visits from a US Census Bureau interviewer. Since 2013, participants can complete ACS questionnaires online. Phone interviews were discontinued in 2017.^13^ Five-year Public Use Microdata Samples (PUMS) are meant to represent current population estimates. In the ACS, there were 1 892 255 children. Of these 1 848 056 were US-born white, 25 557 were US-born MENA, 12 614 were foreign-born white, and 6028 were foreign-born MENA children.

### Measures

#### Independent variables

The independent variable was created by combining variables on race, ethnicity, and place of birth. For NHIS, participants selected from flashcards which included the minimum reporting standards to measure race (eg, White, Black, or African American, Asian) (National Center for Health Statistics, 2019; Office of Management and Budget, 1997) during the in-person interview. To measure ethnicity, the interviewer asked participants if they were Hispanic or Latino/a. To measure place of birth, participants were asked if they were born in the US (including military bases and US territories). Participants who were not born in the US were asked to specify where they were born. To maintain confidentiality, responses were categorized into 10 regions of birth (eg, Middle East, Africa, Europe, Russia/former USSR, among others). We combined responses to race, ethnicity, and region of birth variables to compare US-born non-Hispanic Whites to racial/ethnic foreign-born groups. Specifically, we included foreign-born non-Hispanic Whites from Europe (including Russia/former USSR) and non-Hispanic MENA individuals. The foreign-born MENA category was comprehensive of non-Hispanic White participants who were born in the “Middle East” or “Africa” regions based on previous research.^14^

For ACS, the independent variable was a combined measure of race, ethnicity, place of birth, and ancestry. Race and ethnicity were measured in accordance with OMB guidelines like the NHIS. To measure place of birth, the ACS asked, “where was this person born?” US-born participants checked the box for “in the United States” and listed the state where they were born. Foreign-born participants checked the box “Outside the United States” and printed the name of the foreign country where they were born. To determine ancestry, participants were asked to indicate their ancestry or ethnic origin. Participants could enter up to two ancestry groups. Based on previous studies, adults who reported at least 1 of 43 Arab ancestries located in the Middle East or were born in Comoros, Djibouti, or United Arab Emirates were included.^14-18^ To obtain a more inclusive representation of MENA individuals, we also included individuals who were born in the following MENA countries; Iran, Iraq, Israel, Jordan, Kuwait, Lebanon, Saudi Arabia, Syria, Turkish, Yemen, Algeria, Egypt, Libya, Morocco, Somalia, and Tunisia. Race, ethnicity, place of birth and ancestry were combined to create four categories: US-born non-Hispanic White; foreign-born non-Hispanic White; US-born MENA, and foreign-born MENA.

#### Dependent variables

Three dependent variables were created for health insurance coverage. It was conceptualized as uninsurance (yes or no), public (yes or no), and private (yes or no). The NHIS asks participants whether they or any individuals in the family are “covered by any kind of health insurance or some other kind of health care plan.” Individuals who report “yes” to having any coverage are asked the kind of health insurance coverage they have. Possible responses include private health insurance from their employer, Medicare, Medicaid, Children’s Health Insurance Program, single service plans, among others. Similar questions and responses were collected for the ACS. The ACS asks whether each person in the household is currently covered by insurance from: (a) a current or former employer or union; (b) a policy purchased directly from insurance company; (c) Medicare; (d) Medicaid or any other government program for disability or low income; (e) TRICARE or other military coverage; (f) Veteran’s Administration (VA); (g) Indian Health Service; or (h) any other plans. Individuals respond yes or no to each insurance coverage option.

The main dependent variable was dichotomous indicating *no* health insurance coverage. Children *without* any health insurance coverage were compared to those with any private or public health insurance coverage reported (any private or public coverage). The secondary dependent variables were two dichotomous variables representing (1) private and (2) public health insurance coverage. Children with private health insurance were compared to children without private health insurance (private or no private coverage). Children with public health insurance, inclusive of Medicaid, Children’s Health Insurance Coverage, or any other government programs were compared to children without any public health insurance (private or no coverage).

### Statistical Analysis

Weighted percentages and standard errors were used to describe sociodemographic and other characteristics of children. Multivariable logistic regression was used to test the association between region of birth among non-Hispanic Whites (independent variable) and health insurance coverage (dependent variable). Foreign-born MENA, US-born MENA, and foreign-born White children were compared to US-born non-Hispanic White children as the reference group. US-born non-Hispanic White children were chosen as the reference group given that is the reference group used by other investigators and so that we findings from the current study can be compared to other studies. Data were analyzed using SAS 9.4 survey procedures to account for the sophisticated weighting and complex sample design based on both surveys’ analytic guidelines.^13-19^

## Results

### Sample Characteristics

Selected characteristics of the sample are presented in Table 1. In both the NHIS and ACS data, foreign-born white children were slightly older (9.4 years in the NHIS and 10.8 years in the ACS) compared to foreign-born MENA and US-born whites. Approximately 44% and 67% of foreign-born MENA lived below the poverty level compared to a range of 27% and 39% in other groups.

**Table 1.** Selected Characteristics of Non-Hispanic White and MENA Children, 2000 to 2018 National Health Interview Survey and 2015 to 2019 American Community Survey.

	NHIS			*P*	ACS				*P*
	USB White n = 305 454	FB White n = 4738	FB MENA n = 1769		USB White n = 1.848056	FB White n = 12 614	USB MENA n = 25 557	FB MENA n = 6028	
Age *Mean (SE)*	8.7 (0.02)	9.4 (0.11)	9.1(0.18)	<.0001	8.7 (0.00)	10.8 (0.05)	8.0 (0.04)	10.2 (0.08)	<.0001
Sex				.0191					.0726
Male	51.3 (0.15)	50.0 (0.98)	50.2 (1.57)		51.3 (0.00)	52.0 (0.01)	50.9 (0.00)	52.9 (0.01)	
Female	48.7 (0.15)	50.0 (0.98)	49.8 (1.57)		48.7 (0.00)	48.0 (0.01)	49.1 (0.00)	47.1 (0.01)	
Federal poverty level				<.0001					<.0001
<200%	36.1 (0.33)	33.9 (1.31)	44.7 (1.08)		27.1 (0.00)	31.5 (0.01)	38.9 (0.00)	67.6( 0.01)	
≥200%	63.9 (0.33)	66.1 (1.31)	38.2 (2.44)		72.9 (0.00)	68.5 (0.01)	61.1(0.00)	32.4 (0.01)	

Abbreviations: FB, foreign-born; MENA, Middle Eastern and North African; USB, US-born.

### Age- and Sex-Adjusted Estimates

Age- and sex-adjusted estimates of health insurance coverage are presented in Table 2. In both the NHIS and ACS, foreign-born MENA were more likely to be uninsured or have public health insurance compared to other groups (NHIS = 13.8%; ACS = 9.9%). The foreign-born MENA population was more likely to have public health insurance (NHIS = 32%; ACS = 59.9%) compared to other groups. In the ACS, 4.1% of US-born MENA were uninsured compared to 3.8% of US-born whites, 9.6% of foreign-born whites, and 9.9% of foreign-born MENA.

**Table 2.** Age and Sex-adjusted Estimates of Health Insurance Coverage Among Non-Hispanic White and MENA Children, 2000 to 2018 National Health Interview Survey and 2015 to 2019 American Community Survey.

	National Health Interview Survey (NHIS)			American Community Survey (ACS)		
	Uninsured*	Public*	Private*	Uninsured*	Public*	Private*
US-born White	7.6 (0.00)	15.6 (0.00)	71.8 (0.00)	3.8 (0.00)	29.6 (0.00)	71.1 (0.00)
Foreign-born White	11.0 (0.00)	11.1 (0.01)	70.8 (0.01)	9.6 (0.00)	27.2 (0.01)	65.9 (0.01)
US-born MENA	NA	NA	NA	4.1 (0.00)	40.1 (0.00)	59.1 (0.00)
Foreign-born MENA	13.8 (0.02)	32.0 (0.03)	47.2 (0.03)	9.9 (0.01)	59.9 (0.01)	34.2 (0.01)

Abbreviations: NA, not applicable; MENA, Middle East and North African.

* All *P*’s < .05.

### Multivariable Results

Multivariable logistic regression results in comparison to US-born White children are presented in Table 3. In both the NHIS and ACS, foreign-born MENA (OR = 1.94; 95% CI = 1.48-2.54) and foreign-born White (OR = 1.49; 95% CI = 1.27-1.75) children had higher odds of being uninsured compared to US-born non-Hispanic White children. Results remained statistically significantly different after adjusting for age, sex, and federal poverty level in model 3. When examining public health insurance coverage in the adjusted model (model 3), foreign-born White children were less likely to report having public health insurance coverage compared to US-born non-Hispanic White children. Foreign-born MENA (in the NHIS and ACS) and US-born MENA (in the ACS) were more likely (for example for foreign-born MENA, OR = 1.63; 95% CI = 1.51-1.77) to have public health insurance coverage compared to US-born non-Hispanic White children. Lastly, foreign-born White, US-born MENA and foreign-born MENA children were anywhere from 14% (foreign-born white: OR = 0.86; 95%CI = 0.81-0.91) to 58% (foreign-born MENA: OR = 0.42; 95%CI = 0.38-0.45) less likely to have private health insurance coverage compared to US-born White children.

**Table 3.** Logistic Regression Results Comparing Foreign-born White and Foreign-born MENA to US-born Non-Hispanic White Children, 2000 to 2018 National Health Interview Survey and 2015 to 2019 American Community Survey.

	National Health Interview Survey (NHIS)			American Community Survey (ACS)		
	Model 1	Model 2^a^	Model 3^b^	Model 1	Model 2^a^	Model 3^b^
Uninsured						
US-born White (ref)	1.00	1.00	1.00	1.00	1.00	1.00
Foreign-born White	1.49 (1.27, 1.75)	1.49 (1.27, 1.75)	1.56 (1.30, 1.86)	2.74 (2.52, 2.97)	2.70 (2.49, 2.94)	2.61 (2.40, 2.84)
US-born MENA	NA	NA	NA	1.09 (0.99, 1.19)	1.09 (1.00, 1.20)	1.00 (0.91, 1.10)
Foreign-born MENA	1.94 (1.48, 2.54)	1.94 (1.48, 2.54)	1.50 (1.10, 2.05)	2.84 (2.54, 3.18)	2.82 (2.52, 3.16)	2.11 (1.88, 2.37)
Public						
US-born White (ref)	1.00	1.00	1.00	1.00	1.00	1.00
Foreign-born White	0.66 (0.56, 0.77)	0.67 (0.57, 0.79)	0.64 (0.53, 0.79)	0.84 (0.80, 0.88)	0.89 (0.85, 0.94)	0.72 (0.68, 0.77)
US-born MENA	NA	NA	NA	1.63 (1.58, 1.68)	1.60 (1.55, 1.65)	1.32 (1.27, 1.36)
Foreign-born MENA	2.50 (1.97, 3.18)	2.54 (1.99, 3.23)	1.65 (1.25, 2.18)	3.38 (3.17, 3.61)	3.56 (3.33, 3.80)	1.63 (1.51, 1.77)
Private						
US-born White (ref)	1.00	1.00	1.00	1.00	1.00	1.00
Foreign-born White	0.97 (0.86, 1.09)	0.95 (0.85, 1.07)	0.87 (0.76, 1.00)	0.84 (0.80, 0.88)	0.79 (0.75, 0.83)	0.86 (0.81, 0.91)
US-born MENA	NA	NA	NA	0.58 (0.56, 0.60)	0.59 (0.57, 0.61)	0.70 (0.67, 0.72)
Foreign-born MENA	0.36 (0.29, 0.44)	0.35 (0.29, 0.43)	0.49 (0.39, 0.63)	0.22 (0.21, 0.24)	0.21 (0.20, 0.23)	0.42 (0.38, 0.45)

Abbreviation: NA, not applicable; MENA, Middle Eastern and North African; ref, reference.

a Adjusts for age (continuous) and sex (male referent).

b Adjusts for model 2+ income based on federal poverty level (200% or greater referent).

Multivariable logistic regression results comparing foreign-born MENA children to US-born MENA children (ACS only) are presented in Table 4. Foreign-born MENA children had 2.62 times higher odds (95% CI = 2.26-3.02) of being uninsured compared to US-born MENA children. Results remained statistically significantly different after adjusting for age and sex in model 2 (OR = 2.58; 95% CI = 2.23-2.98) and federal poverty level in model 3 (OR = 2.11; 95% CI = 1.83-2.45). When examining public health insurance coverage and in the adjusted model (model 3), foreign-born MENA children had lower odds of having public health insurance coverage compared to US-born MENA children. Lastly, foreign-born MENA children had 40% lower odds (95% CI = 0.55-0.66) of private health insurance coverage compared to US-born MENA children after adjusting for age, sex, and federal poverty level (model 3).

**Table 4.** Logistic Regression Results Comparing Foreign-born MENA to US-born MENA Children, 2015 to 2019 American Community Survey.

	Model 1	Model 2^a^	Model 3^b^
Uninsured			
US-born MENA (ref)	1.00	1.00	1.00
Foreign-born MENA	2.62 (2.26, 3.02)	2.58 (2.23, 2.98)	2.11 (1.83, 2.45)
Public			
US-born MENA (ref)	1.00	1.00	1.00
Foreign-born MENA	2.08 (1.93, 2.23)	2.23 (2.07, 2.40)	1.24 (1.13, 1.35)
Private			
US-born MENA (ref)	1.00	1.00	1.00
Foreign-born MENA	0.39 (0.36, 0.42)	0.36 (0.33, 0.39)	0.60 (0.55, 0.66)

Abbreviation: MENA, Middle Eastern and North African; ref, reference.

a Adjusts for age (continuous) and sex (male referent).

b Adjusts for model 2+ income based on federal poverty level (200% or greater referent).

## Discussion

### Main Findings

The aims of this study were to estimate and compare the proportion of foreign-born MENA children *without* health insurance to foreign-born White children, US-born White children, and US-born MENA children. Our main finding was that *foreign-born MENA children were more likely to be uninsured compared to US-born White and US-born MENA children.* Our secondary findings were that foreign-born MENA children were more likely to have public insurance and less likely to have private health insurance compared to US-born White and US-born MENA children. These trends persisted when controlling for sociodemographic variables.

This is the first national study to estimate health insurance coverage for MENA children; therefore, we cannot compare our findings to other studies. Our study is consistent with other research studies that demonstrate foreign-born children overall, and within all races and ethnicities, including non-Hispanic Whites, have higher rates of being uninsured compared to US-born children.^20^ Our study delves deeper and disaggregates non-Hispanic White children to elucidate that this profile persists for foreign-born MENA children.

The research literature about MENA children and their health insurance status is sparse. In an unpublished scoping review, the authors identified approximately 700 empirical articles. Sixty-nine of these articles focused on the health of MENA children; and only eight articles reported on MENA children’s health insurance coverage, but none of them analyzed national data that provides baseline estimates. This is the first national study to estimate health insurance coverage for MENA children in the US.

### Strengths and Limitations

A strength of this study was the use of the only two nationally representative data sources to capture health-related information among MENA children. While these surveys have been used for measuring health outcomes among MENA adults,^6,14,16-18^ this is one of the few studies to use both data sources to measure health outcomes among MENA children.^3^

Our study goes beyond previous studies by including foreign-born MENA children born in North Africa and expanding the ancestry variable to include Arab, non-Arab, and trans-national ancestries in the MENA region instead of limiting it to Arab ancestries only. Both surveys allowed us to describe health insurance coverage among foreign-born MENA children by measuring region of birth while only the ACS allowed for estimates among US-born MENA children by also measuring ancestry. The differences in age- and sex-adjusted estimates for US-born White uninsured children (7.6% uninsured NHIS, 3.8% uninsured ACS) highlight the need to identify US-born MENA children from the US-born White category to improve the validity of the estimates for White children. This is not only important for MENA and White group comparisons but for other comparisons between US-born White and other racial/ethnic groups (Black, Hispanic, and Asian). This key finding contributes to recent calls by leading MENA researchers to disaggregate MENA individuals from the White group to elucidate disparities within the White population and improve scientific integrity for health disparities research.^21,22^

The study had a few limitations. Our health insurance outcomes variables were self-reported which may limit the validity of our findings. However, previous population-based research has shown that self-reported health insurance information is reliable when compared to administrative reports^23^; therefore, we do not think this potential information bias affected our results. Another limitation is the smaller sample size of MENA children compared to other racial and ethnic groups. However, the data are weighted and generalizable to the MENA population in the US, which addresses the issue of sample size, but limits the potential of including other variables and controlling for potential confounders. If we controlled for additional variables, the odds ratios and confidence intervals might be unstable. A third limitation is that the ACS does not directly measure personal health information (ie, health behaviors and conditions). It measures only health insurance coverage. If the ACS measured personal health information, we would have compared the results between the NHIS and ACS. A final limitation is that the NHIS and ACS follow different sampling methods, which is one of the reasons the ACS had a larger sample size for MENA compared to the NHIS. While this is overcome with weighting strategies, it is important to recognize this difference might have affected the findings.

It is well-established that health insurance coverage predicts health care access and use and influences diagnosis and treatment of health conditions. Our findings contribute to the dearth of literature in the maternal and child health field examining disparate health insurance coverage among MENA children. Our findings also have implications for public and commercial insurance companies dedicated to reducing racial and ethnic disparities in health insurance coverage and health outcomes among children. Strategies such as updating enrollment forms for both public and private health insurance plans to appropriately capture race, ethnicity, and nativity can aid in identifying and monitoring key disparities among MENA children. Future studies should design, implement, and evaluate interventions aimed at reducing disparities in health insurance coverage among MENA children. A systematic review demonstrated that the following types of interventions may increase enrollment of children in public or private health insurance plans: (1) community health advocates who provide health insurance information and negotiate with insurers; and (2) disseminating insurance applications in hospital emergency departments.^24^ Others have advocated for expanding and making Children’s Health Insurance Program (CHIP) permanent.^25^

Lastly, our findings align with the Centers for Medicare & Medicaid Services (CMS) health equity framework, which focuses on 4 priority areas.^26^ These are: (1) expand the collection, reporting, and analysis of standardized data; (2) assess causes of disparities within CMS programs and address inequities in policies and operations to close gaps; (3) build capacity of health care organizations and the workforce to reduce health and health care disparities; and (4) advance language access, health literacy, and the provision of culturally tailored services. The current study is the first of its kind to estimate health insurance status among MENA children using nationally representative data. The findings are a call to action to meet the CMS’ priority areas and the Office of Management and Budget’s most recent proposal.

In January 2023, the Office of Management and Budget published proposals with a request for public comment on how they are considering making changes to how race and ethnicity data are collected in the US on the decennial census and other federal forms.^27^ During the three-month public comment period that includes a specific request for public comments on the addition of a MENA checkbox on the US Census and other federal forms, overwhelming support was given for the addition of a separate checkbox from the White race group. Among 3355 comments that were reviewed thus far, most mentioned the addition of a MENA checkbox (71%) of which 98% supported. Among those that supported having a separate checkbox, over half (55%) mentioned the need for a MENA checkbox specifically due to the need for language and linguistic services to be provided to this heterogeneous population.^28,29^ An appropriate and relevant ethnic identifier that will allow individuals of MENA backgrounds to be counted will contribute to the health equity framework of CMS and many other similar initiatives.
